# How do we measure unmet need within sexual and reproductive health? A systematic review

**DOI:** 10.1177/17579139221118778

**Published:** 2022-09-20

**Authors:** D Solomon, M Cabecinha, J Gibbs, F Burns, CA Sabin

**Affiliations:** Institute for Global Health, University College London, Gower Street, London WC1E 6BT, UK; Institute for Global Health, University College London, London, UK; Institute for Global Health, University College London, London, UK; Institute for Global Health, University College London, London, UK; Royal Free London NHS Foundation Trust, London, UK; Institute for Global Health, University College London, London, UK

**Keywords:** sexual health, rep, health inequalities

## Abstract

**Background:**

Addressing health inequality with sexual and reproductive health requires an understanding of unmet need within a range of populations. This review examined the methods and definitions that have been used to measure unmet need, and the populations most frequently assessed.

**Methods:**

Five databases (PubMed, Web of Science, Scopus, The Cumulative Index to Nursing and Allied Health Literature (CINAHL) and Health Management and Policy Database (HMIC)) were searched for studies that described quantitative measurement of unmet need within sexual and/or reproductive health between 2010 and 2021. A narrative synthesis was then undertaken to ascertain themes within the literature.

**Results:**

The database search yielded 19,747 papers; 216 papers were included after screening. 190 studies assessed unmet reproductive health need, of which 137 were analyses of trends among people living in low/lower-middle income countries; 181 used cross-sectional data, with only nine analyses being longitudinal. Eighteen studies analysed unmet sexual health need, of which 12 focused on high and upper-middle income populations. 16 papers used cross-sectional analyses. The remaining 10 studies examined unmet need for a combination of sexual and reproductive health services, eight among populations from upper-middle or high income countries. All were cross-sectional analyses. 165 studies used the Demographic and Health Surveys (DHS) definition of unmet need; no other standardised definition was used among the remaining papers.

**Discussion:**

There is a significant focus on unmet need for contraception among women in low income countries within the published literature, leaving considerable evidence gaps in relation to unmet need within sexual health generally and among men in particular, and unmet reproductive health need in high income settings. In addition, using an increased range of data collection methods, analyses and definitions of unmet need would enable better understanding of health inequality in this area.

## Introduction

There is a large burden of sexual and reproductive morbidity across the globe, a burden that disproportionately affects some of the world’s most vulnerable groups.^1^ This pattern of illness and inequality is likely to be attributable, at least in part, to a combination of unmet needs.^2^ It is, however, difficult to define, characterise or measure unmet need within healthcare,^3^ and there are currently very few systems in place that identify needs within sexual and reproductive health, and monitor whether those needs are being met. Although unmet need for contraception has been measured repeatedly across a range of populations,^4^ there is much less discourse within the published literature regarding unmet need within reproductive health more broadly, or unmet need within sexual health. In addition, there has been little analysis of the methods that are being used to identify unmet need, and whether these methods are appropriately identifying the needs of the populations most at risk.

This review is a systematic investigation of the trends within the published literature surrounding unmet need in sexual and reproductive health (SRH) over the past 11 years. In particular, this review will examine the methods that have been used to characterise and measure unmet need, the populations in which unmet need within reproductive and sexual health has been most frequently measured, and the definitions of unmet need that have been used within these analyses.

## Methods

### Search strategy

This review was undertaken according to the Preferred Reporting Items for Systematic Reviews and Meta-Analyses (PRISMA) guidelines. To ensure a thorough review of the literature, a search of five databases was undertaken: PubMed, Web of Science, Scopus, The Cumulative Index to Nursing and Allied Health Literature (CINAHL) and the Health Management and Policy Database (HMIC). Studies that described a quantitative method to elucidate levels of unmet need within sexual and/or reproductive health in a specific population were included in the literature review. Exclusion criteria were studies that were not in English, systematic reviews and studies that used entirely qualitative methods (although mixed-methods studies were included). Maternity care was excluded from the definition of reproductive health for the purposes of this review. The search period was 2010 to 2021–in part for ease of analysis, due to the broad search strategy, and in part because methods described prior to 2010 were likely to be out of date, particularly if they had not been used again in subsequent, more recent, studies.

### Study selection

Three stages of study selection were used to identify papers for inclusion within this literature review. Two reviewers (DS and MC) used Covidence software to assign 20% of titles identified during the database search for inclusion or exclusion. Any discrepancies were discussed between reviewers until there was 100% concordance, and DS then assigned the remaining titles. This process was repeated for the abstracts of the papers that had been flagged for inclusion during the title round. Once all abstracts had been screened, DS screened the full text of the papers that had been flagged for inclusion, and selected the papers that would proceed to data extraction.

### Data extraction

A data extraction form was created in Microsoft Excel, and this was used to record relevant data from the remaining studies. The data extraction process captured whether the study concerned sexual or reproductive health, the sub-topic of interest, the country of data collection, the geographical level of analysis (multinational, national or regional), the income status of the setting (high, upper-middle, lower-middle or low income), the population of interest, the type of study, the methods used, the definition of unmet need and the source of this definition. The nature of the research question (ascertaining trends in the measurement of unmet need within sexual and reproductive health), and the heterogeneity of the included studies, meant that meta-analysis was an inappropriate methodology for analysis of the extracted data. A narrative synthesis of the themes within the literature was therefore carried out in accordance with the Synthesis without meta-analysis (SWiM) PRISMA extension guidance.^5^

## Results

**Figure 1.**
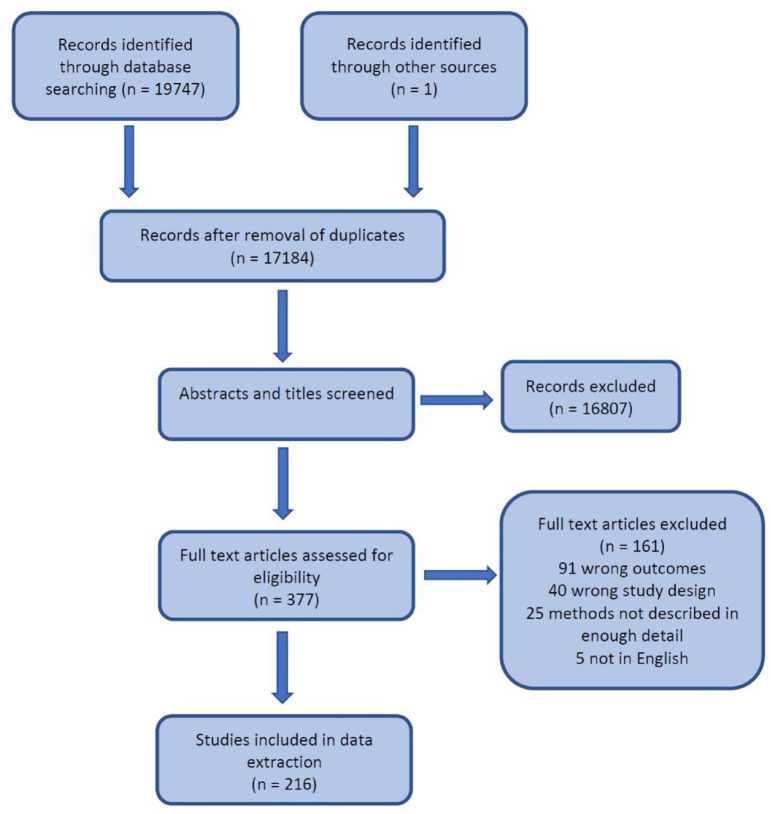
PRISMA flow diagram.

The database search yielded 19,747 papers (Figure 1), and one paper was added after a search of the grey literature. 17,184 remained after removal of duplicates, and 377 remained after screening of abstracts and titles. The full text of these articles was subsequently screened; 91 were removed due to outcomes that did not relate to unmet need or SRH, 40 were removed due to study design (i.e. studies that did not attempt to calculate unmet need), 25 were removed as the methods were not described in enough detail, and five were removed as they were not in English. Data were subsequently extracted from the remaining 216 papers. The entire list of papers can be found summarised in Supplemental Appendices 1, 2 and 3.

### Reproductive health

The majority of the studies found during this literature review (190 out of 216) were analyses of unmet need within reproductive health (Box 1).

**Box 1:** Summary box 1: reproductive health

• Literature predominantly focused on unmet need for contraception among women in low and lower-middle income countries. • Most common definition of unmet need: Westoff and Bradley definition used in the Demographic and Health Surveys. • Data most commonly collected using questionnaires. • Analyses were predominantly cross-sectional secondary analyses of routinely collected data.

#### Methods

The most commonly used method of data collection was the utilisation of questionnaire data. Nearly all of the studies collected information using questionnaires (179 out of 190) – seven studies reviewed medical records, two used modelling analyses, one used focus groups, and one used spatial epidemiology techniques.^6^ Almost all of the analyses (*n* = 181) were cross-sectional, with the other nine being longitudinal. The high prevalence of certain methodologies was at least partially due to the fact that a large proportion of the papers were secondary analyses of similar datasets. Fifty-one of the 190 papers that focused on reproductive health used secondary analyses of data from the Demographic and Health Surveys (DHS) – a series of nationally representative household surveys that are conducted once every five years in 90 low and middle income countries – while another 23 used data from other national health surveys that use similar methodology to the DHS.

#### Population

Most of the studies were analyses of trends among populations living in low or lower-middle income countries; these comprised 137 papers, compared to 51 that were based on populations from upper-middle and high income countries, and two papers that aimed to perform global comparisons. Half of the papers (*n* = 95) drew conclusions at the national or multinational level, with the other half concentrating on regional analyses.

Only six papers considered the contraceptive needs of men. The remaining 184 papers focused solely on unmet need among women, with 89 limiting their analyses to women of reproductive age (usually defined as 15–45 years); 50 of these papers only analysed trends among women of this age group who were married or in-union.

#### Definition of unmet need

Among the 190 analyses of unmet need for contraception, 165 used the same definition of unmet need – the Westoff and Bradley indicator that is used as part of the DHS (or a slightly modified version). According to this definition, women are considered to have unmet need if they report being fecund and sexually active, would like to stop or postpone childbearing, and are not currently using a modern contraceptive method.^7^

Outside of these papers, definitions of unmet need were diverse. Only one study – a household questionnaire study analysing unmet need for contraception among married women in Mali and Benin^8^ – utilised a measure of perception. Women were defined as having perceived met need (compared to real met need) if they were using an ineffective method of contraception. Five other questionnaire-based studies defined unmet need for contraception as a discordance between desired method or source of contraceptives and the actual method that was currently being used.^9–13^ Two papers used disparity between underserved groups and a defined baseline to define unmet need; a UK-based study compared contraceptive use and abortion rates between women suffering from opioid addiction and the general population,^14^ and a Dutch study analysed the disparity between contraceptive counselling and prescription among refugee women, other migrant women and native Dutch women. Two studies (one in Australia,^15^ one in Ethiopia)^16^ defined unmet need as lack of postpartum contraception planning. The outcomes used to measure unmet reproductive need outside of the need for contraception were equally varied. The three papers that analysed unmet need for cervical screening measured lack of uptake of routine cervical screening^17–19^ and similarly, the analysis of unmet need for HPV vaccination measured women in the appropriate age group who had not received the vaccine during the Australian catch-up programme.^20^ A cross-sectional analysis of unmet need for abortion services in Ghana defined any woman who reported an abortion outside of a facility as having unmet need.^21^ Two studies analysed unmet need for abortion at the facility level, one defining unmet need as the inability of a health service to provide appropriate abortion services to women seeking treatment^22^ and one using the treatment rate for complications of induced abortion as a marker of unmet need.^23^ A study in Ireland investigated unmet need for abortion by comparing demand for services pre- and postlegalisation.^24^ An analysis of unmet need in India defined women as having an unmet need if they had suffered from a reproductive morbidity and either sought care from a qualified medical practitioner but did not complete treatment; sought treatment from an unqualified practitioner; engaged in home remedy or did not seek any treatment.^25^ Three studies used geographical techniques to measure unmet need: one measuring the correlation between driving distance from an abortion service and the geographical abortion rate,^26^ one defining women who had travelled across country borders to access abortion as having unmet need^27^ and one mapping ‘contraception deserts’ (areas with no affordable family planning clinic within a reasonable driving distance) within the US.^6^

### Sexual health

Compared to those focusing on reproductive health, significantly fewer studies within this review analysed unmet need within sexual health (*n* = 18) (Box 2).

**Box 2:** Summary box 2: sexual health

• Literature predominantly focused on unmet need among women in higher and upper-middle countries. • Range of definitions of unmet need. • Data most commonly collected using questionnaires. • Analyses were predominantly cross-sectional analyses of primary data.

#### Methods

Methods of analysing unmet need within sexual health followed a similar pattern to analyses of unmet need within reproductive health; 13 of the 18 papers used questionnaire data, and 16 analyses were cross-sectional. The five papers that did not use questionnaire data used a diverse range of methods – three papers used medical records review, one used modelling techniques to estimate unmet need and one compared demand for sexual health services before and after an intervention. Unlike the analyses of unmet need within reproductive health, no papers used secondary data analyses to estimate unmet need for sexual health; 17 papers used primary data collection, and one used routinely collected data from national data sets.

#### Population

Compared to analyses of unmet need within reproductive health, papers that examined unmet need within sexual health analysed a range of populations. Twelve papers focused on high and upper-middle income populations, and six looked at populations from low and lower-middle income countries. The majority (*n* = 13) drew conclusions at the regional level, with four being national analyses and one being a multinational analysis. Only one used a nationally representative cohort, with the other papers concentrating on defined subgroups: people attending genitourinary medicine (GUM) clinics, female sex workers (FSW), men who have sex with men (MSM), incarcerated women, adolescent psychiatric patients, foreign-born HIV patients, men and women under the age of 25, university students and people seeking care for gynaecological cancers.

#### Definition of unmet need

The definitions of unmet need used within these analyses were equally diverse. Five analyses^28–32^ defined unmet need as non-utilisation of sexual health services despite STI symptoms or history of unsafe sex. Another UK analysis measured unmet need by asking attendees at one of seven GUM clinics whether they had been previously turned away,^33^ while two analyses of similar UK populations measured both provider delay (the gap between first contact with a health service and access to treatment) and patient delay (the gap between start of symptoms and seeking care).^34,35^ The two analyses of access to sexual health services outside of the GUM setting (in an adolescent psychiatric unit^36^ and a gynaecological oncology unit)^37^ used lack of sexual health counselling within medical notes as an indicator of unmet need, and an analysis of foreign-born Europeans used a negative HIV test in the years prior to an HIV diagnosis as an indicator of unmet need for HIV prevention services.^38^ A Canadian study used the change in demand for STI services after the implementation of a women’s healthcare centre within a prison as an indicator of unmet need,^39^ and an Australian analysis of routinely collected data defined unmet need as the gap between estimated chlamydia incidence and actual chlamydia diagnoses.^40^ A study in Papua New Guinea defined individuals who had fallen through gaps in the 90-90-90 cascade as having unmet need for HIV prevention or treatment.^41^ The four studies investigating unmet need for PrEP all used different definitions: non-use of PrEP despite eligibility,^42^ disparity between regional PrEP use and regional STI prevalence,^43^ new HIV infection while waiting for inclusion in a PrEP trial,^44^ and increased PrEP demand after reduction in the cost of PrEP.^45^

### Sexual and reproductive health

Ten of the studies found during this literature review examined unmet need for a combination of sexual and reproductive health services within a certain population (Box 3).

**Box 3:** Summary box 3: sexual and reproductive health

• Literature predominantly focused on unmet need among women in higher and upper-middle countries. • Range of definitions of unmet need. • Data most commonly collected using questionnaires. • Analyses were predominantly cross-sectional analyses of primary data.

#### Methods

All 10 studies investigating unmet need in sexual and reproductive health used questionnaire data: eight studies analysed primary data and two were secondary analyses of data from larger national studies. All 10 analyses were cross-sectional.

#### Population

Eight of the studies that examined unmet need in sexual and reproductive health were undertaken among populations from upper-middle or high income countries. Seven drew conclusions at the regional (rather than national or multinational) level. There was, once again, a focus on population subgroups, with only one study (a South African household study) collecting data from all eligible people over the age of 15.

#### Definition of unmet need

The definitions of unmet need for sexual and reproductive health care varied between papers. Two studies used a range of definitions: both used the Westoff and Bradley definition of unmet need for contraception, never having had a Pap smear and symptoms consistent with STIs that had remained untreated as indicators of unmet need.^46,47^ A cross-sectional household questionnaire study conducted in China measured unmet need among older women by asking about untreated STI symptoms and intrauterine device (IUD) retention after the menopause.^48^ One analysis compared SRH service use between women who reported similar sexual activity but differing levels of religious participation.^49^ One study examined the disparity in SRH demand between areas that provided youth-friendly services and those that did not.^50^ Three studies included measures of perceived need,^51–53^ and two measured unmet need by asking participants if they had received the SRH services that they felt they needed.^50,54^

## Discussion

This literature review outlined 216 studies published over the past 11 years that examined unmet need in a range of populations using a variety of methods. Despite this heterogeneity, a number of patterns emerged on closer analysis that gave some insight into the way that unmet need within sexual and reproductive health is conceptualised, and revealed numerous gaps in the literature.

### Topic

Most of the studies within this literature review were on the subject of unmet need within reproductive health, and within these, the majority focused on unmet need for contraception. Some of the reasons for this are likely historical; widespread discourse surrounding the concept of unmet need within sexual and reproductive health largely began in the 1960s within the ‘family planning’ space,^4^ meaning that the definitions and methodology used in this area have shaped the way that unmet need is conceptualised within both theoretical and implementation science, to the point where ‘unmet need for family planning’ is used as a key indicator by the United Nations without much discussion of unmet need in other areas of sexual and reproductive health.^55^ Another reason for the prevalence of studies that measure unmet need for contraception is likely to be feasibility. Unmet need for contraception is easier to define and measure due to the presence of a defined endpoint – unplanned pregnancy – that has few other causes. Measuring unmet need in sexual health is far more challenging. Tying a specific need to an outcome within sexual health is made difficult by the lack of data from those who are not receiving care, and causal links between needs and outcomes are less clearly defined. There remains, however, a large and under-treated global burden of morbidity within sexual health,^56^ indicating that the conceptualisation and measurement of unmet need within sexual health should also be a research priority.

### Population

The majority of the studies within this review aimed to measure unmet need among cisgender women – this trend that was particularly apparent among studies that were on the topic of unmet need within reproductive health. Although the reproductive needs of women are often more immediately apparent, there was a paucity of discourse within the literature about the role of unmet need for contraception among cisgender men with regards to unplanned pregnancy; something that is likely to become increasingly relevant as efforts to expand the range of male contraceptives continue.^57^ There was also very little discussion of the needs of gender-diverse populations, and the needs of transgender women were often grouped together with the needs of MSM. Given the recognised morbidities and barriers to care faced by gender-diverse populations,^58^ this is a significant gap in the literature exploring unmet need within sexual and reproductive health.

A large proportion of studies concentrated on the needs of women of reproductive age (usually defined as 15–45 years), and among these papers, a significant majority limited analysis to women who were married or in a union similar to marriage. This was in part due to the high prevalence of data from household studies, particularly those carried out via the DHS, that often specifically ask questions regarding reproductive health to women within this age group. Most studies that limited analyses to married or in-union women explained this as a method of confirming that respondents are sexually active. This assumption, however, may be somewhat archaic – as marriage rates decrease^59^ and the age of first marriage increases globally^60^ while age of sexual debut remains relatively steady,^61^ the needs of an increasing number of women are not being measured. In addition, these methods overlook the needs of groups such as sex workers and those who have same-sex partners, who are likely to have unmet sexual and reproductive needs that lie outside of the bounds of a monogamous heterosexual relationship.^62^ In addition, the focus on women of childbearing age leaves a gap in the understanding of the sexual and reproductive health needs of those who are younger than 15 years or older than 45 years, two groups who have been demonstrated to experience unique patterns of sexual and reproductive morbidity.^63,64^

Among studies that analysed unmet need within reproductive health, the majority investigated populations within low and lower-middle income areas. This trend was reversed among papers that investigated sexual health and SRH, the majority of which analysed populations within upper-middle and high income countries. There appear to be two resultant gaps in the literature. There is little investigation of unmet need within reproductive health in high income countries, despite the inequalities in reproductive outcomes that have been identified in these settings.^65,66^ Similarly, there is little investigation of unmet need within sexual health in low income countries, despite the recognised lack of appropriate sexual health services in many such settings.^67^

### Methods

Questionnaire studies were particularly prevalent within this literature review, and were used to examine unmet need within both reproductive and sexual health. Although such methods are often useful, the fact that questionnaires are the primary method used for the assessment of unmet need within sexual and reproductive health inherently leaves some areas of enquiry neglected. Questionnaires, particularly those centred around potentially sensitive topics, are susceptible to both recall bias – in which one group is systematically more likely to remember certain events, and social desirability bias – in which respondents are systematically more likely to report behaviours or opinions that they think will be viewed favourably.^68^ In addition, the interpretation of a concept as complex as unmet need can be dependent on the perspective of the researcher. A 2017 mixed-methods study found that the perceptions of stakeholders did not at all mirror the responses of the local population when both were asked about the drivers of unmet need for contraception.^69^ Despite this, very few studies directly asked respondents about their perception of need, or about demand.

A large proportion of the studies in this review were secondary analyses of large household studies. Only one of these studies – the National Survey of Sexual Attitudes and Lifestyles – was specifically designed to investigate sexual and reproductive health at the population level. The other surveys are focused on health more generally, and therefore may not be the most useful tools for investigating unmet need within sexual and reproductive health. In addition, the DHS is designed for monitoring and evaluation of national programme goals,^4^ and the fact that it is one of the main sources of information regarding global unmet need within reproductive health means that there is little understanding within the published literature of the drivers of unmet need or the differences between regions or subgroups.

## Strengths and Limitations

It is important to acknowledge the limitations of this review. The inclusion criteria for this review did not include qualitative analyses, which limits the discourse within this article to quantitative measures of unmet need. The role of qualitative and mixed-methods work within this area is a topic that would benefit from exploration in the future. The literature search was also limited to papers that were in English, which may have resulted in the omission of relevant literature. We believe, however, that the breadth of the search is likely to have captured the majority of the papers within this area.

This article also has multiple strengths. This is, to our knowledge, the first systematic review to examine the methodology being used to calculate unmet need within sexual and reproductive health across the published literature. The breadth and international scope of this review have allowed the authors to conduct an in depth analysis of the measurement of unmet need in a range of settings, allowing for a broader understanding of a concept that is vital within public health.

## Conclusions

This review revealed multiple gaps in our understanding of unmet need within sexual and reproductive health. The vast majority focus on unmet need for contraception among in-union women in low income countries, leaving a significant need for investigation of unmet need within sexual health, unmet reproductive health need in high income settings and unmet need among women who are not of reproductive age. In addition, there is a need for data collected using a range of methods that can reflect regional patterns and sub-group trends and begin to elicit the causes of unmet need. If these gaps are not addressed, we run the risk of repeatedly measuring unmet need within sexual and reproductive health but not collecting the data that will allow us to make significant and sustainable change.

## Supplemental Material

sj-pdf-1-rsh-10.1177_17579139221118778 – Supplemental material for How do we measure unmet need within sexual and reproductive health? A systematic review Supplemental material, sj-pdf-1-rsh-10.1177_17579139221118778 for How do we measure unmet need within sexual and reproductive health? A systematic review by D Solomon, M Cabecinha, J Gibbs, F Burns and CA Sabin in Perspectives in Public Health

sj-pdf-2-rsh-10.1177_17579139221118778 – Supplemental material for How do we measure unmet need within sexual and reproductive health? A systematic review Supplemental material, sj-pdf-2-rsh-10.1177_17579139221118778 for How do we measure unmet need within sexual and reproductive health? A systematic review by D Solomon, M Cabecinha, J Gibbs, F Burns and CA Sabin in Perspectives in Public Health

sj-pdf-3-rsh-10.1177_17579139221118778 – Supplemental material for How do we measure unmet need within sexual and reproductive health? A systematic review Supplemental material, sj-pdf-3-rsh-10.1177_17579139221118778 for How do we measure unmet need within sexual and reproductive health? A systematic review by D Solomon, M Cabecinha, J Gibbs, F Burns and CA Sabin in Perspectives in Public Health
