# Supplementary material for: How do we measure unmet need within sexual and reproductive health? A systematic review
Source: Perspect Public Health. 2022 Sep 20;144(2):78–85. doi: 10.1177/17579139221118778 (PMC10916345; doi:10.1177/17579139221118778)
Supplement: sj-pdf-1-rsh-10.1177_17579139221118778 – Supplemental material for How do we measure unmet need within sexual and reproductive health? A systematic review [file sj-pdf-1-rsh-10.1177_17579139221118778.pdf]

Study ID	Country	Scope	Income status	Sub-category	Setting	Population	Type of study	Methods	Data source
Abdella 2013	Ethiopia	National	Low income	Abortion	Health facility	Women seeking treatment for abortion complications	Cross-sectional	Medical records review	Primary collection
Abeje 2016	Ethiopia	National	Low income	Contraception	Health facility	reproductive age on ART	Cross-sectional	Questionnaire	Primary collection
Acacio-Claro 2010	Philippines	National	Low income	Contraception	Household	Women of reproductive age who are either married or in a sexual relationship	Cross-sectional	Questionnaire	2000 Reproductive Health-Family Planning Baseline Surveys
Acera 2017	Spain	National	High income	Cervical screening	GP clinic	Women aged 30 - 70	Cohort	Medical records review	Primary collection
Achwoka 2018	Kenya	National	Lower-middle income	Contraception	MCH clinic	Women attending 6-week or 9-month immunization visits	Cross-sectional	Questionnaire	Primary collection
Adebowale 2014	Burkina Faso	Regional	Low income	Contraception	Household	Married women aged 15 - 49	Cross-sectional	Questionnaire	2010 DHS

Study ID	Country	Scope	Income status	Sub-category	Setting	Population	Type of study	Methods	Data source
Adedini 2015	Nigeria	National	Lower-middle income	Contraception	Household	Women aged 15 - 49	Cross-sectional	Questionnaire	DHS 2008
Ahinkorah 2020	Multiple	Multinational	Low/lower-middle income	Contraception	Household	Married or cohabiting women aged 15 - 24	Cross-sectional	Questionnaire	DHS 2010 - 2018
Ahmed 2013	Pakistan	National	Lower-middle income	Contraception	Household	Women aged 15 - 49	Cross-sectional	Questionnaire	DHS 2006
Ajong 2016	Cameroon	Regional	Lower-middle income	Contraception	Household	Sexually active, in union women aged 15 - 49	Cross-sectional	Questionnaire	Primary collection
Akintade 2011	Lesotho	Regional	Lower-middle income	Contraception	University	Female undergraduate students	Cross-sectional	Questionnaire	Primary collection
Alaba 2015	Nigeria	National	Lower-middle income	Contraception	Household	Women aged 15 - 49	Cross-sectional	Questionnaire	DHS 2013
Alem 2020	Ethiopia	National	Low income	Contraception	Household	Sexually active women aged 15 - 49	Cross-sectional	Questionnaire	DHS 2016
Ali 2013	Sudan	Regional	Low income	Contraception	Household	Ever-married women aged 15 - 49	Cross-sectional	Questionnaire	Primary collection

Study ID	Country	Scope	Income status	Sub-category	Setting	Population	Type of study	Methods	Data source
Almonte 2019	Zambia	National	Lower-middle income	Contraception	Household	Sexually active women aged 15 - 49	Cross-sectional	Questionnaire	DHS 2013
Aptekman 2014	Canada	Regional	High income	Contraception	Refugee health clinic	Women aged 15 - 49	Cross-sectional	Questionnaire	Primary collection
Asad 2019	Vietnam	National	Lower-middle income	Contraception	Household	Married/in union women aged 15 - 49	Cross-sectional	Questionnaire	Multiple Indicator Cluster Surveys 2000 - 2014
Asif 2019	Pakistan	Regional	Lower-middle income	Contraception	Household	Married women aged 15 - 49	Cross-sectional	Questionnaire	DHS 2012
Asif 2019	Pakistan	National	Lower-middle income	Contraception	Household	Married women aged 15 - 49	Cross-sectional	Questionnaire	DHS 2012
Asresie 2020	Ethiopia	National	Low income	Contraception	Household	Married women aged 15 - 49	Cross-sectional	Questionnaire	DHS 2016
Atchison 2019	Ethiopia, Nigeria, Tanzania	Regional	Low income	Contraception	Household	Married women aged 15 - 19	Cross-sectional	Questionnaire	Primary collection
Atiglo 2018	Ghana	National	Lower-middle income	Contraception	Household	Women aged 15 - 24 who were fecund, non-pregnant and had ever had sex	Cross-sectional	Questionnaire	DHS 2014

Study ID	Country	Scope	Income status	Sub-category	Setting	Population	Type of study	Methods	Data source
Atiglo 2019	Ghana	National	Lower-middle income	Contraception	Household	Women aged 15 - 49	Cross-sectional	Questionnaire	DHS 2015
Austin 2015	Nigeria	National	Lower-middle income	Contraception	Household	Married women aged 15 - 49	Cross-sectional	Questionnaire	DHS 2003, 2008, 2013
Ayuningtyas 2015	Indonesia	Regional	Upper-middle income	Contraception		Married women aged 15 - 49	Cross-sectional	Questionnaire	Primary collection
Bamgboye 2016	Nigeria	National	Lower-middle income	Contraception	Household	Women aged 15 - 49	Cross-sectional	Questionnaire	National HIV/AIDS and Reproductive Health Survey
Banougnin 2018	Benin	Regional	Lower-middle income	Contraception	Household	Married women aged 15 - 49	Cross-sectional	Questionnaire	DHS 2012
Bawah 2019	Ghana	Regional	Lower-middle income	Contraception	Household	Married women aged 15 - 49	Cross-sectional	Questionnaire	Primary collection
Bellizi 2015	Multiple	Multinational	Low income	Contraception	Household	Women aged 15 - 49	Cross-sectional	Questionnaire	DHS 2005 - 2011

Study ID	Country	Scope	Income status	Sub-category	Setting	Population	Type of study	Methods	Data source
Best 2021	Australia	Regional	High income	Contraception	Clinic	Women who had given birth during the study period while receving social support	Cross-sectional	Medical records review	Primary collection
Bhanderi 2010	India	National	Upper-middle income	Reproductive morbidity	Household	Ever-married women aged 15 - 49	Cross-sectional	Questionnaire	Primary collection
Bhattathiry 2014	Sri Lanka	National	Low income	Contraception	Household	Married women aged 15 - 49	Cross-sectional	Questionnaire	Primary collection
Bhusal 2018	Nepal	Regional	Lower-middle income	Contraception	Household	Married women aged 15 - 49	Cross-sectional	Questionnaire	Primary collection
Bishwajit 2017	Bangladesh	National	Low income	Contraception	Household	Mothers aged between 13 and 49	Cross-sectional	Questionnaire	DHS 2011
Borges 2018	Brazil	Regional	Upper-middle income	Contraception	GP clinic	Women aged 15 - 44 at any stage in pregnancy	Longitudinal	Questionnaire	Primary collection

Study ID	Country	Scope	Income status	Sub-category	Setting	Population	Type of study	Methods	Data source
Bose 2019	Multiple	Multinational	Low income	Contraception	Household	Women aged 15 - 49	Cross-sectional	Questionnaire	DHS 2000 - 2015
Brandao 2019	Mozambique	National	Low income	Cervical screening	Health facility	Women aged 15 - 64	Cross-sectional	Questionnaire	Primary collection
Bras 2021	Ireland	National	High income	Abortion	Clinic	People contacting online telemedicine abortion service	Cross-sectional	Medical records review	Primary collection
Callahan 2014	Bangladesh	National	Low income	Contraception	Household	Married women under the age of 50	Longitudinal	Questionnaire	Bangladesh Microcredit and Health Study
Cammock 2017	New Zealand	Regional	High income	Contraception	Community	Women over the age of 18 who identified as iTaukei	Cross-sectional	Questionnaire	Primary collection
Canfell 2015	Australia	Regional	High income	vaccination (catch up	Household	Women aged 26 - 29	Cross-sectional	Questionnaire	Primary collection
Cannon 2018	USA	Regional	High income	Contraception	Womens' prison	Women 18 - 50	Cross-sectional	Questionnaire	Primary collection

Study ID	Country	Scope	Income status	Sub-category	Setting	Population	Type of study	Methods	Data source
Casterline 2014	45 countries	Multinational	Lower-middle income	Contraception	Household	Women aged 15 - 49	Cross-sectional	Questionnaire	DHS, World Fertility Surveys, Reproductive Health Surveys and Pan-Arab
Catalao 2020	Ethiopia	Regional	Low income	Contraception	Clinic	Women between 18 and 49 who were in the 3rd trimester of pregnancy	Cross-sectional	Questionnaire	Primary collection
Chauhan 2018	India	Regional	Lower-middle income	Contraception	Household	Currently married women who had at least one living child below 10 years of age	Cross-sectional	Questionnaire	Primary collection
Cornford 2015	UK	Regional	High income	Contraception	GP clinic	Women who were receiving treatment for opioid addiction	Cohort	Medical records review	Primary collection

Study ID	Country	Scope	Income status	Sub-category	Setting	Population	Type of study	Methods	Data source
Creanga 2011	Multiple	Multinational	Low income	Contraception	Household	Women aged 15 - 49	Cross-sectional	Questionnaire	DHS 1996 - 2007
Darroch 2013	Global	Multinational		Contraception	Household	Women aged 15 - 49	Cross-sectional	Use of DHS/MICS data to extrapolate at a population level	DHS 2003, 2008, 2012
Das 2021	Myanmar and Phillipines	National	Lower-middle income	Contraception	Household	Women aged 15 - 49	Cross-sectional	Questionnaire	DHS 2015-2017
de Pokomandy 2019	Canada	National	High income	Cervical screening	Community	with HIV aged 16 or older	Cross-sectional	Questionnaire	Women's Sexual and
Debelew 2021	Ethiopia	National	Low income	Contraception	Household	Women aged 15 - 24	Cross-sectional	Questionnaire	PMA 2020
Decat 2011	China	Regional	Upper-middle income	Contraception	Worksite	Women aged 18 - 29	Cross-sectional	Questionnaire	Primary collection
DeGraff 2015	Sri Lanka	National	Lower-middle income	Contraception	Household	Ever-married women aged 15 - 49	Cross-sectional	Questionnaire	DHS 1987 and 2007
Dejene 2021	Ethiopia	Regional	Low income	Contraception	Clinic	Married women on ART aged 15 - 49	Cross-sectional	Questionnaire	Primary collection

Study ID	Country	Scope	Income status	Sub-category	Setting	Population	Type of study	Methods	Data source
Delbiso 2014	Ethiopia	National	Low income	Contraception	Household	Married women aged 15 - 49	Cross-sectional	Questionnaire	DHS 2011
Deyessa 2017	Ethiopia	National	Low income	Contraception	Household	Married women aged 15 - 49	Cross-sectional	Questionnaire	DHS 2016
Dingeta 2019	Ethiopia	Regional	Low income	Contraception	Community	Married women aged 15 - 24	Cross-sectional	Questionnaire	Primary collection
Doctor 2013	Nigeria	National	Lower-middle income	Contraception	Household	Women aged 15 - 49	Cross-sectional	Questionnaire	Mid-Term Household Survey
Doherty 2018	Botswana	Regional	Low income	Contraception	Health facility	Pregnant women over 18	Cross-sectional	Questionnaire	Primary collection
Dowerah 2020	India	Regional	Upper-middle income	Contraception	Household	Married women aged 15 - 49	Cross-sectional	Questionnaire	Primary collection
Dugg 2020	India	Regional	Upper-middle income	Contraception	Clinic	Women aged 18 - 49 living with HIV	Cross-sectional	Questionnaire	Primary collection
Dulli 2016	Rwanda	National	Low income	Contraception	MCH clinic	Women who were at least 6 months post-partum	Cluster RCT	Questionnaire	Primary collection
Edietah 2018	Cameroon	Regional	Lower-middle income	Contraception	Household	Women aged 15 - 49 in a union	Cross-sectional	Questionnaire	Primary collection
Elweshahi 2017	Egypt	Regional	Lower-middle income	Contraception	Health facility	Women attending MMR immunization visits	Cross-sectional	Questionnaire	Primary collection

Study ID	Country	Scope	Income status	Sub-category	Setting	Population	Type of study	Methods	Data source
Embafrash 2019	Ethiopia	Regional	Low income	Contraception	MCH clinic	Postpartum married women of reproductive age in their first year after delivery	Cross-sectional	Questionnaire	Primary collection
Ettarh 2012	Kenya	National	Lower-middle income	Contraception	Household	Women aged 15 - 49	Cross-sectional	Questionnaire	DHS 2008
Fagbamigbe 2018	Nigeria	National	Lower-middle income	Contraception	Household	Married/in union women aged 15 - 49	Cross-sectional	Questionnaire	DHS 2013
Feyissa 2014	Ethiopia	Regional	Low income	Contraception	Health facility	Married/in union women aged 15 - 49	Cross-sectional	Questionnaire	Primary collection
Feyissa 2020	Ethiopia	Regional	Low income	Contraception	Clinic	Women aged 18 - 49 living with HIV	Cross-sectional	Questionnaire	Primary collection
Freitas Goes 2020	Brazil	Regional	Upper-middle income	Abortion	Clinic	Women admitted to hospital for abortion-related care	Cross-sectional	Questionnaire	Primary collection
G/Meskel 2021	Ethiopia	Regional	Low income	Contraception	Household	Married women aged 15 - 49 years	Cross-sectional	Questionnaire	Primary collection

Study ID	Country	Scope	Income status	Sub-category	Setting	Population	Type of study	Methods	Data source
Ganle 2016	Ghana	Regional	Lower-middle income	Abortion	Household	Women aged 15 - 49	Cross-sectional	Questionnaire	Primary collection
Garnsey 2021	England and Wales	National	High income	Abortion	Clinic	Women who had travelled from a different country to access abortion services	Cross-sectional	Questionnaire	Primary collection
Geary 2016	UK	National	High income	Contraception	Household	Heterosexually active men and women aged 16-24	Cross-sectional	Questionnaire	Primary collection
Geary 2016	UK	National	High income	Contraception	Household	Heterosexually active men and women aged 16-24	Cross-sectional	Questionnaire	Primary collection
Gelagay 2015	Ethiopia	Regional	Low income	Contraception	HIV clinic	Married women of reproductive age attending for care at ART clinic	Cross-sectional	Questionnaire	Primary collection
Genet 2015	Ethiopia	Regional	Low income	Contraception	Household	Married women aged 15 - 49	Cross-sectional	Questionnaire	Primary collection
Gichangi 2021	Kenya	National	Lower-middle income	Contraception	Household	Married women aged 15 - 49	Cross-sectional	Questionnaire	PMA 2020

Study ID	Country	Scope	Income status	Sub-category	Setting	Population	Type of study	Methods	Data source
Groene 2021	Ethiopia	National	Low income	Contraception	Household	Women 18 - 49	Cross-sectional	Questionnaire	PMA 2020
Guure 2019	Ghana	National	Lower-middle income	Contraception	Household	Married/in union women aged 15 - 49	Cross-sectional	Questionnaire	DHS 2014
Hailemariam 2011	Ethiopia	National	Low income	Contraception	Household	Married women aged 15 - 49	Cross-sectional	Questionnaire	DHS 2000 and 2005
Hellstrom 2019	Sweden	National	High income	Contraception	Household	Women aged 16 - 49	Cross-sectional	Questionnaire	Primary collection
Hossain 2016	USA	National	High income	Contraception	Household	Men aged 15 - 44 who have ever had sex	Cross-sectional	Questionnaire	National Survey of Family Growth 2006 - 2010
Inci 2020	Germany	Regional	High income	Contraception	Community	Women aged 14 - 74 who were registered refugees	Cross-sectional	Questionnaire	Primary collection
Islam 2016	Bangladesh	National	Low income	Contraception	Household	Married women aged 13–24 years	Cross-sectional	Questionnaire	Bangladesh DHS 2011
Jain 2014	Pakistan	National	Lower-middle income	Contraception	Household	Married women	Longitudinal	Questionnaire	Primary collection

Study ID	Country	Scope	Income status	Sub-category	Setting	Population	Type of study	Methods	Data source
Jhangri 2012	Uganda	Regional	Low income	Contraception	HIV clinic	Women aged 18–44 years, married or cohabitating with a partner, with an HIV test result	Cross-sectional	Questionnaire	Primary collection
Johnson 2012	Ghana	National	Lower-middle income	Contraception	Household	Women aged 15 - 49	Cross-sectional	Small area estimation techniques	DHS 2003 and Ghana Population and Housing Census (GPHC) 2000
Juarez 2018	Mexico	National	Upper-middle income	Contraception	Household	Women aged 15 - 49	Cross-sectional	Questionnaire	National Survey of Demographic Dynamics
Kantorova 2021	185 countries	International	International	Contraception	Household	Married/in union women aged 15 - 19	Cross-sectional	Modelling	Multiple household surveys
Kassie 2021	Ethiopia	Regional	Low income	Contraception	Clinic	Women aged 15 - 49 living with HIV	Cross-sectional	Questionnaire	Primary collection
Kebede 2019	Ethiopia	Regional	Low income	Contraception	Community	Married women aged 15 - 49	Cross-sectional	Questionnaire	Primary collection

Study ID	Country	Scope	Income status	Sub-category	Setting	Population	Type of study	Methods	Data source
Khalil 2017	Saudi Arabia	National	High income	Contraception	Household	Married women aged 15–49 years	Cross-sectional	Questionnaire	Primary collection
Khan 2009	Madagascar	Regional	Low income	Contraception	Health facility	Sex workers aged 15 - 55 years	Cross-sectional	Questionnaire	Primary collection
Komowasa 2021	India	Regional	Upper-middle income	Contraception	Household	Married women aged 15 - 49	Cross-sectional	Questionnaire	National Family Health Survey (2005 and 2015)
Kreitzer 2021	USA	National	High income	Contraception	Clinic	n/a	Cross-sectional	GIS	Primary collection
Leon 2014	India	Regional	Upper-middle income	Contraception	Household	Married women of reproductive age	Cross-sectional	Questionnaire	Primary collection
Letamo 2014	Botswana	National	Upper-middle income	Contraception	Household	Married/in union women aged 15 - 49	Cross-sectional	Questionnaire	Botswana Family Health Survey 2007
Leyser-Whalen 2011	USA	Regional	High income	Contraception	Publicly funded reproductive health clinics	Women aged 16 - 24	Cross-sectional	Questionnaire	Primary collection
Li 2020	103 countries	National	Low and middle income	Contraception	Household	Women aged 15 - 34	Cross-sectional	Questionnaire	DHS 2000 - 2017

Study ID	Country	Scope	Income status	Sub-category	Setting	Population	Type of study	Methods	Data source
Liauw 2016	Canada	Regional	High income	Contraception	Womens' prison	Incarcerated women aged 18 - 49	Cross-sectional	Questionnaire	Primary collection
Lim 2014	China	Regional	Upper-middle income	Contraception	Community	20 who reported transactional sex in the past 6 months	Cross-sectional	Questionnaire	Primary collection
Long 2019	Kenya	Regional	Lower-middle income	Contraception	Community	over who were living with HIV and had recently engaged in transactional sex	Cross-sectional	Questionnaire	Primary collection
Luo 2021	China	National	Upper-middle income	Contraception	Clinic	Women who had recived an induced abortion	Cross-sectional	Medical records review	Primary collection
Lutalo 2018	Uganda	Regional	Low income	Contraception	Household	Women aged 15 - 49	Longitudinal	Questionnaire	Primary collection
Machiyama 2014	Ghana	National	Lower-middle income	Contraception	Household	Women aged 15 - 49	Cross-sectional	Questionnaire	DHS 1988, 1993, 1998, 2003, 2008
Mahoro 2018	Rwanda	National	Low income	Contraception	Household	Married women aged 15 - 49	Cross-sectional	Questionnaire	DHS 2005, 2010, 2014
Malqvist 2018	Nepal	National	Lower-middle income	Contraception	Household	Married/in union women aged 15 - 49	Cross-sectional	Questionnaire	Multiple Indicator Cluster Survey 2014

Study ID	Country	Scope	Income status	Sub-category	Setting	Population	Type of study	Methods	Data source
Mehata 2014	Nepal	National	Lower-middle income	Contraception	Household	Women aged 15–49 years who had a birth in the last 24 months	Cross-sectional	Questionnaire	DHS 2011
Mehata 2020	Nepal	National	Lower-middle income	Contraception	Household	Married women aged 15 - 49	Cross-sectional	Questionnaire	DHS 2016
Mekonnen 2011	Ethiopia	Regional	Low income	Contraception	Household	Women aged 15 - 49	Cross-sectional	Questionnaire	Primary collection
Mochache 2018	Kenya	Regional	Lower-middle income	Contraception	Household	Women aged 18 - 45	Cross-sectional	Questionnaire	Primary collection
Mohanty 2009	India	Regional	Lower-middle income	Contraception	Household	Currently married women 15-39	Longitudinal	Questionnaire	Primary collection
Moore 2015	Multiple	Multinational	Low income	Contraception	Household	Women aged 15–49 years who had a birth in the last 0–23 months	Cross-sectional	Questionnaire	DHS 2005 - 2012
Moreau 2019	46 countries	Multinational	Low and middle income	Contraception	Household	Women aged 15 - 49	Cross-sectional	Questionnaire	DHS 2010 - 2018
Muhoza 2009	Rwanda	National	Low income	Contraception	Household	Women aged 15 - 49 in a union	Cross-sectional	Questionnaire	DHS 2005

Study ID	Country	Scope	Income status	Sub-category	Setting	Population	Type of study	Methods	Data source
Mukherjee 2021	India	Regional	Upper-middle income	Contraception	Household	Married women aged 15 - 49	Cross-sectional	Questionnaire	Primary collection
Mulenga 2020	Zambia	National	Lower-middle income	Contraception	Household	Married women aged 15 - 49	Cross-sectional	Questionnaire	DHS 2013/14
Nkoka 2020	Malawi	National	Low income	Contraception	Household	Sexually active women aged 15 - 49	Cross-sectional	Questionnaire	DHS 2015
Nyauchi 2014	Kenya	National	Lower-middle income	Contraception	Household	Non-pregnant women aged 15 - 49	Cross-sectional	Questionnaire	DHS 2008
Nzokirishaka 2018	Burundi	National	Low income	Contraception	Household	Married women aged 15 - 49	Cross-sectional	Questionnaire	DHS 2010
Odland 2021	Malawi	Regional	Low income	Contraception	Clinic	Pregnant women attending antenatal clinic	Cross-sectional	Questionnaire	Primary collection
Oginni 2015	Nigeria	National	Low income	Contraception	Household	Women aged 15 - 49	Longitudinal	Questionnaire	Nigeria DHS 2003, 2008, 2013
Ogunjuyigbe 2010	Nigeria	Regional	Lower-middle income	Contraception	Household	Men and women aged over 18	Cross-sectional	Questionnaire	Primary collection
Olagbuji 2021	South Africa	Regional	Upper-middle income	Contraception	Clinic	Girls living with HIV aged 14 -17	Cross-sectional	Questionnaire	Primary collection

Study ID	Country	Scope	Income status	Sub-category	Setting	Population	Type of study	Methods	Data source
Owens 2020	USA	Regional	High income	Contraception	Community	Women aged 18 - 49 who had transactional sex and injected drugs in the last year	Cross-sectional	Questionnaire	National HIV Behavioral Surveillance Survey
Ozedemir 2019	Turkey	Regional	Upper-middle income	Contraception	Household	Married women aged 15 - 49	Cross-sectional	Questionnaire	Primary collection
Pack 2014	Liberia	Regional	Low income	Contraception	Community	Sexually experienced women aged 14 - 25	Cross-sectional	Questionnaire	Primary collection
Packer 2020	Afghanistan	Regional	Low income	Contraception	Community	Married men aged 18 -49 years	Cross-sectional	Questionnaire	Primary collection
Paregallo 2011	Haiti	National	Low income	Contraception	Household	Women aged 15 - 49	Cross-sectional	Questionnaire	DHS 2006
Pasha 2015	India, Pakistan, Zambia, Kenya, Guatemala	Multinational	Low and middle income	Contraception	Household	Women who were 42 days post partum	Cross-sectional	Questionnaire	Primary collection
Patra 2015	India	Regional	Upper-middle income	Contraception	Household	Ever-married women aged 15 - 49	Cross-sectional	Questionnaire	DLHS-3

Study ID	Country	Scope	Income status	Sub-category	Setting	Population	Type of study	Methods	Data source
Pearson 2014	Benin, Burkina Faso, Mali	Multinational	Low income	Contraception	Household	Women aged 15 - 49 and their partners	Cross-sectional	Questionnaire	DHS 2001 - 2006
Pham 2020	Papua New Guinea	Regional	Lower-middle income	Contraception	Household	Women aged 15 - 49	Cross-sectional	Questionnaire	Integrated Health and Demographic Surveillance System
Potter 2014	USA	Regional	High income	Contraception	MCH clinic	women aged 18–44 who wanted to delay childbearing for at least 24 months	Cohort	Questionnaire	Primary collection
Prasad 2016	India	Regional	Upper-middle income	Contraception	Household	Women aged 15 - 49	Cross-sectional	Questionnaire	Primary collection
Prusty 2014	India	Regional	Upper-middle income	Contraception	Household	Ever-married women aged 15 - 49	Cross-sectional	Questionnaire	DLHS 2007-2008
Puri 2021	Nepal	Regional	Lower-middle income	Contraception	Health facility	All women who gave birth in a participating hospital during study period	Longitudinal	Questionnaire	Primary collection
Raben 2018	The Netherlands	Regional	High income	Contraception	GP clinic	Women aged 15 - 49	Cohort	Medical records review	Primary collection

Study ID	Country	Scope	Income status	Sub-category	Setting	Population	Type of study	Methods	Data source
Rai 2017	India	Regional	Lower-middle income	Contraception	Household	Married women aged 15 - 49	Cross-sectional	Questionnaire	DLHS 2002 - 2004
Raj 2013	India	Regional	Lower-middle income	Contraception	Household	Currently married women 15-49	Cross-sectional	Questionnaire	District Level Household Survey III (2008)
Rizvi 2020	Cambodia	National	Lower-middle income	Contraception	Household	Sexually active women aged 15 - 29	Cross-sectional	Questionnaire	DHS 2014
Rucinski 2018	South Africa	Regional	Upper-middle income	Contraception	HIV clinic	Non-pregnant, HIV-positive women aged 18–35	Cohort	Questionnaire	Primary collection
Sanchez-Franco 2021	Colombia	National	Upper-middle income	Contraception	Household	Women aged 13 - 49	Cross-sectional	Questionnaire	DHS 2015
Sarnak 2020	Uganda	National	Low income	Contraception	Household	Sexually active women non-contracepting aged 15 - 49	Longitudinal	Questionnaire	PMA 2020
Saya 2021	India	Regional	Lower-middle income	Contraception	Household	Married couples aged between 18 and 49	Cross-sectional	Questionnaire	Primary collection
Shukla 2021	India	National	Lower-middle income	Contraception	Household	Married women aged 15 - 49	Cross-sectional	Questionnaire	NFHS-3 and NFHS-4

Study ID	Country	Scope	Income status	Sub-category	Setting	Population	Type of study	Methods	Data source
Sidibe 2020	Guinea	National	Low income	Contraception	Household	Women aged 15 - 49	Cross-sectional	Questionnaire	DHS 1999, 2005, 2012 and 2018
Simegn 2020	Ethiopia	Regional	Low income	Contraception	University	Female students	Cross-sectional	Questionnaire	Primary collection
Sinai 2017	Mali and Benin	National	Low income	Contraception	Household	Married women of reproductive age	Cross-sectional	Questionnaire	Primary collection
Sinai 2018	Nigeria	National	Lower-middle income	Contraception	Household	Married women under the age of 25 and men who are married to women under the age of 25	Cross-sectional	Questionnaire	Primary collection
Sinai 2019	Nigeria	Regional	Lower-middle income	Contraception	Household	Married women aged 15 - 49	Cross-sectional	Focus group	Primary collection
Singh 2020	Nepal, India, Bangladesh, Pakistan	National	Lower-middle income	Availabilty of safe abortion and post-abortion care	Health facility	National statistics	Cross-sectional	Questionnaire	Health Facility Surveys
Siswanto 2017	Indonesia	National	Upper-middle income	Contraception	Household	Married women aged 15 - 49 who are up to 24 months postpartum	Cross-sectional	Questionnaire	PMA 2020/DHS 2007 and 2012

Study ID	Country	Scope	Income status	Sub-category	Setting	Population	Type of study	Methods	Data source
Solanke 2019	Nigeria	Regional	Lower-middle income	Contraception	Household	Married women aged 15 - 49	Cross-sectional	Questionnaire	DHS 2008 and 2013
Solomon 2017	Ethiopia	Regional	Low income	Contraception	Household	Married women aged 15 - 49	Cross-sectional	Questionnaire	Primary collection
Sothornwit 2020	Thailand	Regional	Upper-middle income	Contraception	Clinic	Gynaecology patients aged 40-49	Cross-sectional	Questionnaire	Primary collection
Sultan 2010	Egypt	Regional	Lower-middle income	Contraception	Community	Currently married women 15-49	Cross-sectional	Questionnaire	Primary collection
Sultan 2010	Egypt	National	Lower-middle income	Contraception	Household	Married women aged 15 - 49	Cross-sectional	Questionnaire	Primary collection
Sulthana 2015	India	Regional	Lower-middle income	Contraception	Household	Married women aged 15 - 45	Cross-sectional	Questionnaire	Primary collection
Tadele 2019	Ethiopia	National	Low income	Contraception	Household	Non-pregnant women aged 15 - 49	Cross-sectional	Questionnaire	PMA 2020
Takele 2012	Ethiopia	Regional	Low income	Contraception	Household	Married women of reproductive age	Cross-sectional	Questionnaire	Primary collection
Tegegn 2017	Ethiopia	Regional	Low income	Contraception	Household	Women up to one year postpartum	Cross-sectional	Questionnaire	Primary collection

Study ID	Country	Scope	Income status	Sub-category	Setting	Population	Type of study	Methods	Data source
Thompson 2021	USA	Regional	High income	Contraception	Clinic	People seeking abortion within any of 1948 US counties	Cross-sectional	Modelling	Primary collection
Tusubira 2020	Uganda	Regional	Low income	Contraception	Clinic	Women who had given birth within the past two months	Cross-sectional	Questionnaire	Primary collection
Twizelimana 2021	Malawi	Regional	Low income	Contraception	Community	Female sex workers aged between 18 and 49	Cross-sectional	Questionnaire	Primary collection
Uddin 2016	Bangladesh	National	Low income	Contraception	Household	Married women aged 15 - 49 and their spouses	Cross-sectional	Questionnaire	DHS 2007
Verma 2014	India	Regional	Upper-middle income	Contraception	Community	Fecund married women aged 15 - 49	Cross-sectional	Questionnaire	Primary collection
Wafula 2015	Kenya	National	Lower-middle income	Contraception	Household	Married women aged 15 - 49	Cross-sectional	Questionnaire	DHS 2008
Wai 2019	Myanmar	Regional	Lower-middle income	Contraception	Household	Currently married women 18-49	Cross-sectional	Questionnaire	Primary collection
Wai 2019	Myanmar	Regional	Lower-middle income	Contraception	Household	Married women aged 18 -49 years	Cross-sectional	Questionnaire	Primary collection

Study ID	Country	Scope	Income status	Sub-category	Setting	Population	Type of study	Methods	Data source
Wang 2019	Nigeria	National	Lower-middle income	Contraception	Household	Women aged 15 - 49	Cross-sectional	Questionnaire	DHS 2003, 2008, 2013
Wanyenze 2015	Uganda	Regional	Low income	Contraception	HIV clinic	Men and women living with HIV	Cross-sectional	Questionnaire	Primary collection
Wasnik 2013	India	Regional	Upper-middle income	Contraception	Household	Married women of reproductive age	Cross-sectional	Questionnaire	Primary collection
Wassihun 2021	Ethiopia	Regional	Low income	Contraception	Clinic	Women who had given birth at least six weeks previously	Cross-sectional	Questionnaire	Primary collection
Wemakor 2020	Ghana	Regional	Lower-middle income	Contraception	Household	Women of childbearing age (not defined)	Cross-sectional	Questionnaire	Primary collection
White 2020	USA	Regional	High income	Contraception	Clinic	Women 4 - 10 weeks post abortion	Cross-sectional	Questionnaire	Primary collection
Wolde 2020	Ethiopia	Regional	Low income	Contraception	Household	Married women aged 15 - 49	Cross-sectional	Questionnaire	Primary collection
Wood 2021	Burkina Faso, Kenya, Kinshasha (DRC) and Lagos (Nigeria)	National and regional	Low/lower-middle income	Contraception	Household	In union women aged 15 - 49	Cross-sectional	Questionnaire	PMA 2020 (both rounds)/2017 and 2019
Workie 2017	Ethiopia	National	Low income	Contraception	Household	Women aged 15 - 49	Cross-sectional	Questionnaire	Primary collection

Study ID	Country	Scope	Income status	Sub-category	Setting	Population	Type of study	Methods	Data source
Wulifan 2017	Burkina Faso	Regional	Low income	Contraception	Household	Currently pregnant women	Cross-sectional	Questionnaire	Primary collection
Wulifan 2019	Ghana	National	Lower-middle income	Contraception	Household	Married women aged 15 - 49 years	Cross-sectional	Questionnaire	Ghana DHS 2014
Yadav 2009	India	Regional	Upper-middle income	Contraception	Household	Married women aged 15 - 44 and their spouses	Cross-sectional	Questionnaire	Primary collection
Yadav 2021	India	Regional	Upper middle income	Contraception	Household	Married women aged 15 - 24	Cross-sectional	Questionnaire	Primary collection
Yaya 2018	Angola	National	Low income	Contraception	Household	Married women aged 15 - 49	Cross-sectional	Questionnaire	DHS 2015
Yaya 2020	Togo	Regional	Low income	Contraception	Clinic	Women aged 15 - 49 living with HIV	Cross-sectional	Questionnaire	Primary collection
Yaya 2021	Gambia and Mozambique	National	Low income	Contraception	Household	Women aged 15 - 49	Cross-sectional	Questionnaire	DHS 2011 and 2013
Yigzaw 2015	Ethiopia	National	Low income	Contraception	Household	Women aged 15 - 49	Cross-sectional	Questionnaire	DHS 2005 and 2011
Yotebieng 2015	DRC	Regional	Low income	Contraception	HIV clinic	Women living with HIV	Cross-sectional	Questionnaire	Primary collection
Zulhijriani 2020	Burkina Faso	National	Low income	Contraception	Household	Women aged 15 - 49	Cross-sectional	Questionnaire	PMA 2015
