# Supplementary material for: How do we measure unmet need within sexual and reproductive health? A systematic review
Source: Perspect Public Health. 2022 Sep 20;144(2):78–85. doi: 10.1177/17579139221118778 (PMC10916345; doi:10.1177/17579139221118778)
Supplement: sj-pdf-2-rsh-10.1177_17579139221118778 – Supplemental material for How do we measure unmet need within sexual and reproductive health? A systematic review [file sj-pdf-2-rsh-10.1177_17579139221118778.pdf]

Study ID	Country	Scope	Income status	Sub-category	Setting	Population	Type of study	Methods	Data source
Geressu 2012	UK	Regional	High income	Sexual health services	GUM clinic	All new patients attending seven GUM clinics across England	Cross-sectional	Questionnaire	Primary collection
Ghimire 2011	Nepal	Regional	Lower-middle income	Sexual health services	Community	Female sex workers aged 17 - 46	Cross-sectional	Questionnaire	Primary collection
Bowring 2019	Cameroon	Regional	Lower-middle income	Sexual health services	Community	Female sex workers and men who have sex with men who are over 18	Cross-sectional	Questionnaire	Primary collection
Tanton 2017	UK	National	High income	Sexual health services	Household	Men and women aged 16–74 years resident in Britain	Cross-sectional	Questionnaire	Primary collection
Golden 2017	Canada	Regional	High income	STI testing	Health facility	Patients admitted to adolescent psychiatric unit	Cross-sectional	Medical records review	Primary collection
Dunne 2018	UK	Regional	High income	SRH service use	GUM clinic	Patients at one of seven GUM clinics	Cross-sectional	Questionnaire	Primary collection
Fakoya 2018	Multiple	Multinational	High income	HIV prevention services	HIV clinic	People who were HIV-positive, 18 years or older, foreign-born residents and diagnosed within five years of recruitment	Cross-sectional	Questionnaire	Primary collection

Study ID	Country	Scope	Income status	Sub-category	Setting	Population	Type of study	Methods	Data source
Dave 2011	UK	National	High income	Sexual health services	GUM clinic	Three groups: men <25, women < 25, men >25	Cross-sectional	Questionnaire	Primary collection
Kyagba 2014	Uganda	Regional	Low income	Sexual health services	University	Undergraduate university students	Cross-sectional	Questionnaire	Primary collection
Chapman 2018	USA	Regional	High income	Sexual health communication	Health facility	Women who had been treated with radiotherapy for gynaecological cancers	Cross-sectional	Questionnaire	Primary collection
Besney 2018	Canada	Regional	High income	STI testing and Pap smear	Womens' prison	Incarcerated women	Longitudinal	Comparison pre/post implementation of a womens' healthcare centre	Primary collection
Gray 2020	Australia	National	High income	Chlamydia	Routine health data	Men and women aged 15 - 29	Modelling	Modelling	Routinely collected national datasets
Bhatia 2020	Vietnam	Regional	Lower-middle income	PrEP	Community	MSM who were 16 or older	Cross-sectional	Questionnaire	Primary collection

Study ID	Country	Scope	Income status	Sub-category	Setting	Population	Type of study	Methods	Data source
Hakim 2020	Papua New Guinea	Regional	Lower-middle income	HIV	Community	Female sex workers, MSM and transgender women	Cross-sectional	Questionnaire and rapid HIV testing	Primary collection
Hart 2021	Canada	Regional	High income	STBBI prevalence/PrEP	Community	MSM	Cross-sectional	Questionnaire and STI testing	Primary collection
Jewsbury 2021	England	Regional	High income	PrEP	Clinic	Patients awaiting recruitment to a PrEP trial	Cross-sectional	Review of clinical records	Primary collection
Mysior 2020	Germany	National	High income	PrEP	Clinic	PrEP users	Cross-sectional	Review of clinical records	Primary collection
Storm 2020	Nepal	Regional	Lower-middle income	Sexual health services	Community	MSM and transgender women	Cross-sectional	Questionnaire	Primary collection
