# Supplementary material for: How do we measure unmet need within sexual and reproductive health? A systematic review
Source: Perspect Public Health. 2022 Sep 20;144(2):78–85. doi: 10.1177/17579139221118778 (PMC10916345; doi:10.1177/17579139221118778)
Supplement: sj-pdf-3-rsh-10.1177_17579139221118778 – Supplemental material for How do we measure unmet need within sexual and reproductive health? A systematic review [file sj-pdf-3-rsh-10.1177_17579139221118778.pdf]

Study ID	Country	Scope	Income status	Sub-category	Setting	Population	Type of study	Methods	Data source
Ama 2013	Botswana	Regional	Upper-middle income	SRH service use	Household	Women over 50	Cross-sectional	Questionnaire	Primary collection
Hall 2012	USA	National	High income	SRH service use	Household	Women under 25	Cross-sectional	Questionnaire	National Survey of Family Growth
Javadnoori 2018	Iran	Regional	Upper-middle income	SRH service use	Household	men aged 20 - 60	Cross-sectional	Questionnaire	Primary collection
Sun 2014	China	Regional	Upper-middle income	Sexual and reproductive	Household	Women aged 50 - 64	Cross-sectional	Questionnaire	Primary collection
van Rie 2018	South Africa	Regional	Upper-middle income	Sexual and reproductive	Household	Adults over the age of 15	Cross-sectional	Questionnaire	Primary collection
Baroudi 2020	Sweden	National	High income	SRH service use	Language schools	People aged 16 - 29 who were born outside of Sweden	Cross-sectional	Questionnaire	Primary collection
Rose 2021	New Zealand	Regional	High income	SRH service use	Online	People ages 15 -24	Cross-sectional	Questionnaire	Primary collection
Haile 2020	Ethiopia	Regional	Low income	SRH service use	School	People aged 15 - 19	Cross-sectional	Questionnaire	Primary collection

Study ID	Country	Scope	Income status	Sub-category	Setting	Population	Type of study	Methods	Data source
O'Brien 2019	Canada	National	High income	SRH care	Clinic	Women living with HIV accessing HIV care	Cross-sectional	Questionnaire	CHIWOS
Wilson 2020	Kenya	Regional	Lower-middle income	SRH service use	Community	Sexually active girls aged 14-24	Cross-sectional	Questionnaire	Transitions study
